# Treatment of therapy-related acute myeloid leukemia and underlying multiple myeloma with decitabine/venetoclax and daratumumab

**DOI:** 10.1007/s00277-021-04490-3

**Published:** 2021-03-13

**Authors:** Khalid Shoumariyeh, Johannes Jung, Michael Rassner, Sandra Maria Dold, Veronika Riebl, Milena Pantic, Georg Herget, Reinhard Marks, Michael Lübbert, Ralph Wäsch, Monika Engelhardt

**Affiliations:** 1 Department of Hematology, Oncology and Stem Cell Transplantation, Medical Center - University of Freiburg, Faculty of Medicine, University of Freiburg, Hugstetterstr. 53, 79106 Freiburg, Germany; 2 Faculty of Biology, University of Freiburg, Freiburg, Germany; 3 Comprehensive Cancer Center Freiburg (CCCF), Faculty of Medicine, Medical Center - University of Freiburg, Freiburg, Germany; 4 Department of Orthopedics and Trauma Surgery, Faculty of Medicine, Medical Center - University of Freiburg, Freiburg, Germany

Dear Editor,

With increased survival of patients with multiple myeloma (MM), therapy-related myelodysplastic syndrome (t-MDS) and t-acute myeloid leukemia (AML) may occur more frequently [1, 2]. We present here a patient with high-risk (HR) MM, who developed t-MDS and subsequent t-AML. AML treatment with decitabine/venetoclax resulted in complete remission (CR) of the t-AML, while progressive disease of MM was treated with daratumumab. We hypothesize that upregulation of CD38 in bone marrow plasma cells (BMPCs) after decitabine/venetoclax may have enhanced MM response. Additionally, we performed a review of the literature (Suppl. Table 1).

In June 2015, a 64-year-old female was diagnosed with IgG kappa (κ) MM. IgG levels were 46g/L, κ-serum-free light chains (SFLC) 75.4mg/L and ß2-microglobulin 8.2mg/L (Fig. 1A). Anemia with a hemoglobin (Hb) of 8.4g/dL and osteolytic lesions were present. BMPC infiltration was 90%, and fluorescence in situ hybridization (FISH) revealed hyperdiploidy and del17p13 (Fig. 1B (a) and C). The MM was classified as International Staging System (ISS) III, R-ISS III, with 2/4 CRAB criteria. The patient’s revised myeloma comorbidity index was intermediate-fit [3].

**Fig. 1:**
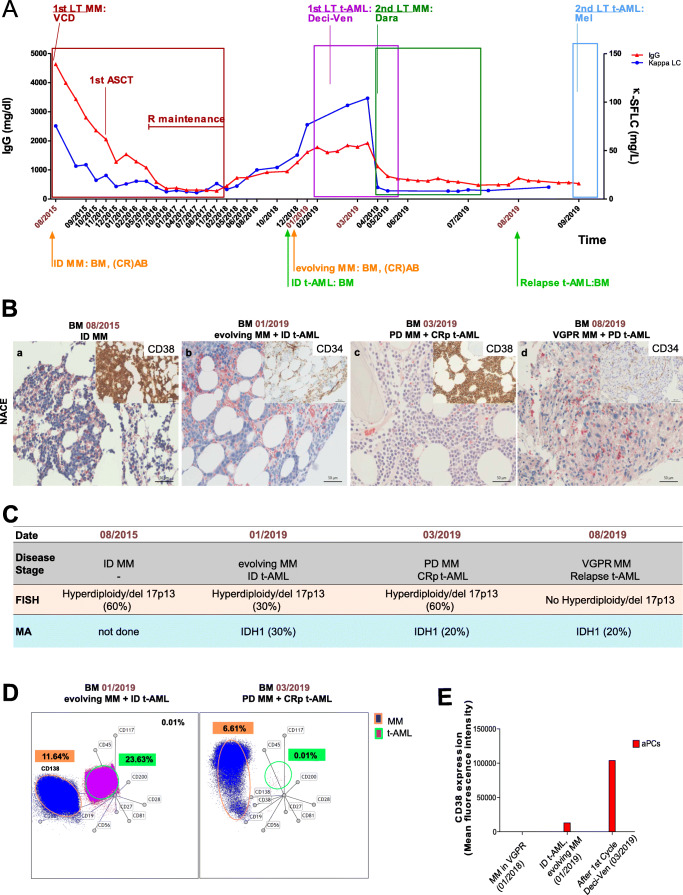
The patient’s clinical, cytogenetic, and molecular results, describing both MM and t-AML clones. **A** Course of serological parameters (IgG and κ-SFLC) and disease state of MM (orange) and t-AML (green) between August 2015 and September 2019. Lines of treatment for each entity are depicted. **B** NACE and immunohistochemical stainings (CD34 or CD38) of BM biopsies from initial diagnosis of MM (a. August 2015), initial diagnosis of t-AML (b. January 2019), after the 1^st^ cycle of decitabine/venetoclax (c. March 2019), and at t-AML relapse (d. August 2019). **C** Remission status (gray), fluorescence in situ hybridization (FISH) analyses (beige), and molecular diagnostics (blue) during the disease course. Percent of cells positive for hyperdiploidy and/or del17p13 as assessed by FISH at initial diagnosis of MM (August 2015), at initial diagnosis of t-AML (January 2019), and before treatment initiation with daratumumab (March 2019). Allele frequency of the *IDH1* mutation at initial diagnosis of t-AML (January 2019), after the 1^st^ cycle decitabine/venetoclax (March 2019), and at t-AML relapse (August 2019). **D** Radar plot of the flow cytometry analysis with the 10-color MFC panel, showing the myeloma (dark blue/orange circle) and leukemia (pink/green circle) population at the initial diagnosis of t-AML (January 2019) and after the 1^st^ cycle of decitabine/venetoclax (March 2019). **E** Mean fluorescence intensity of CD38 expression of aberrant plasma cells (aPCs) in the BM in January 2018, when the MM was in remission, at initial diagnosis of t-AML (January 2019) and after the 1^st^ cycle of decitabine/venetoclax (March 2019). aPC, aberrant plasma cells; ASCT, autologous stem cell transplantation; BM, bone marrow; CRAB, hypercalcemia, renal impairment, anemia, bone lesions; CRp, complete remission with incomplete platelet recovery; Dara, daratumumab; Deci-Ven, decitabine/venetoclax; 1^st^ LT MM, first-line treatment multiple myeloma; 1^st^ LT t-AML, first-line treatment therapy-related acute myeloid leukemia; FISH, fluorescence in situ hybridization; ID, initial diagnosis; IDH1, isocitrate dehydrogenase 1; IgG, immunoglobulin G; LC, light chains; 2^nd^ LT MM, second-line treatment multiple myeloma; Mel, melphalan; MA, molecular analysis; NACE, naphthol-AS-D-chloracetatesterase; PD, progressive disease; R, Lenalidomide; SFLC, serum-free light chains; VCD, bortezomib, cyclophosphamide, dexamethasone; VGPR, very good partial remission

First-line therapy with bortezomib, cyclophosphamide, and dexamethasone was followed by autologous stem cell transplantation and maintenance therapy with lenalidomide (Fig. 1A). After 1½ years (11/2017), lenalidomide was discontinued due to worsening anemia (Hb 10g/dL) and leukopenia (2.8x10^6^/L). BM assessment did not reveal increased PCs or MDS, and serological parameters indicated stable disease.

In January 2019, pancytopenia worsened (Hb 8.4g/dL, leukocytes 0.59x10^6^/L, platelets 12x10^6^/L) and κ-SFLCs increased (Fig. 1A). Another BM biopsy revealed BMPCs of 50% and myeloid blasts of 22% (Fig. 1B (b)). Molecular analyses identified mutations in *DNMT3A* and *IDH1* (Fig. 1C). Coexistence of MM and t-AML (Fig. 1D, left) was confirmed by 10-color multiparameter flow cytometry (MFC) analysis of the BM [4].

Due to frailty at that time, she was ineligible for intensive AML induction therapy. Therefore, treatment with decitabine/venetoclax was started in February 2019. A BM biopsy in March 2019 confirmed CR of the t-AML (Fig. 1B (c)). However, PCs assessed by immunohistochemistry for CD38 had increased to 90%, and MFC confirmed aberrant PCs (aPCs) (Fig. 1D, right); therefore 2^nd^ line daratumumab treatment was initiated (Fig. 1A). This induced VGPR and peripheral blood (PB) counts improved (Hb 10.2g/dL, leukocytes 3.2x10^6^/L, platelets 94x10^6^/L).

In June 2019, after worsening pancytopenia re-emerged and myeloid blasts were detectable in PB smears, decitabine/venetoclax was re-initiated. The BM biopsy in August 2019 showed persisting (30%) immature myeloid blasts (Fig. 1B (d)), upon which melphalan per os was started [5]. The patient died 2 months later of t-AML/MM progression, 50 months after the diagnosis of HR MM, and 9 months after t-AML.

In summary, after decitabine/venetoclax induction and favorable t-AML-response, MM progression required 2^nd^ line daratumumab treatment, resulting in VGPR and improvement of PB counts. Notably, decitabine/venetoclax may have resulted in upregulation of CD38 (Fig. 1 D and E), possibly augmenting the response to daratumumab, although single-cell CD38 expression on aPCs before and after decitabine/venetoclax was not performed. In line with this hypothesis, Choudhry et al. showed that treatment of MM cell lines and primary patient samples with the demethylating agent 5-azacytidine resulted in CD38 upregulation [6]. Moreover, ATRA and the pan-deacetylase-inhibitor panobinostat may increase expression of CD38 in MM [7, 8]. Similarly, Zhao et al. demonstrated upregulation of CD38 on CD8-positive T-cells of AML patients receiving decitabine [9]. Furthermore, daratumumab has been shown to be effective in targeting adult CD38-positive AML and T-cell acute lymphoblastic leukemia (T-ALL) as well as pediatric T-ALL blasts in a preclinical patient-derived xenograft mouse model, and a phase II study (NCT03384654) investigating the efficacy of daratumumab in relapsed and refractory T-ALL is currently ongoing [10, 11]. Recently, Berthon et al. reported about a patient with simultaneous AML and MM who concomitantly received 5-azacytidine and daratumumab during MM relapse (Suppl. Table 1) [12]. Clinical trials are currently under way to investigate whether pretreatment with demethylating agents enhances the efficacy of daratumumab.

## Supplementary Information

ESM 1 (DOCX 58.8 kb).
