# Supplementary material for: Treatment of therapy-related acute myeloid leukemia and underlying multiple myeloma with decitabine/venetoclax and daratumumab
Source: Ann Hematol. 2021 Mar 13;100(6):1637–40. doi: 10.1007/s00277-021-04490-3 (PMC8116235; doi:10.1007/s00277-021-04490-3)
Supplement: Supplementary file 1 — (DOCX 58.8 kb). [file 277_2021_4490_MOESM1_ESM.docx]

**Suppl. Table 1. Review of the literature of selected MM and AML diagnoses with available information about treatment and outcome**

| **#** | **Year of report** | **Age/sex** | **MM** | **AML** | **MM / AML treatment** | **OS (months)** | **Reference** | **Notable findings** |
| --- | --- | --- | --- | --- | --- | --- | --- | --- |
| 1 | 2021 | 68/F | IgGk | t-MDS->t-AML | VCD -> Mel200 -> Len -> Deci/Ven -> Dara | 50 with MM;  9 with t-AML | Shoumariyeh et al. | Sequential MM/t-AML treatment,  upon MM relapse: Dara-mono response after Deci/Ven-therapy |
| 2 | 2020 | 68/M | IgA l | Synchronous MM+AML | 7+3 -> Aza/Len -> Aza/Dara | 55 | Berthon et al.[12] | Concomitant AML/MM treatment after 7+3 induction,  upon MM relapse: Aza/Dara treatment |
| 3-13 | 1977-2017 | 51-79; M:F=8:3 | IgG:IgA=6:3  k:l=6:3 | Synchronous MM+AML  or  MM preceding AML | 7+3: n=3  Flag-IDA: n=1  CAG: n=1  Aza: n=1  MP: n=2  HU+IFN: n=1  SC: n=2  Allo-SCT: n=1 | 1 - 13.5+ | Table 1 in Berthon et al. [12] | In most: short OS due to difficult to treat AML + MM |

**Abbreviations**: MDS, myelodysplastic syndrome; AML, acute myeloid leukemia;, t-AML, therapy-related AML; MM, multiple myeloma; OS, overall survival; VCD, bortezomib, cyclophosphamide, dexamethasone; Len, lenalidomide, Deci/Ven, decitabine/venetoclax; Dara, daratumumab; Aza, azacytidine; FLAG-IDA, fludarabine, idarubicin, G-CSF, high-dose cytarabine; CAG, low-dose cytarabine, aclarubicin, G-CSF; MP, melphalan/prednisone; HU+IFN, hydroxyurea and interferon, SC, supportive care; Allo-SCT, allogeneic stem cell transplantation.
